# A Cost-Effective IoT System for Monitoring Indoor Radon Gas Concentration

**DOI:** 10.3390/s18072198

**Published:** 2018-07-08

**Authors:** Oscar Blanco-Novoa, Tiago M. Fernández-Caramés, Paula Fraga-Lamas, Luis Castedo

**Affiliations:** Department Computer Engineering, Faculty of Computer Science, Universidade da Coruña, 15071 A Coruña, Spain; o.blanco@udc.es (O.B.-N.); luis@udc.es (L.C.)

**Keywords:** IoT, radon, WSN, wireless sensor networks, home automation, domotics, sensors, smart home

## Abstract

Radon is a noble gas originating from the radioactive decay chain of uranium or thorium. Most radon emanates naturally from the soil and from some building materials, so it can be found in many places around the world, in particular in regions with soils containing granite or slate. It is almost impossible for a person to detect radon gas without proper tools, since it is invisible, odorless, tasteless and colorless. The problem is that a correlation has been established between the presence of high radon gas concentrations and the incidence of lung cancer. In fact, the World Health Organization (WHO) has stated that the exposure to radon is the second most common cause of lung cancer after smoking, and it is the primary cause of lung cancer among people who have never smoked. Although there are commercial radon detectors, most of them are either expensive or provide very limited monitoring capabilities. To tackle such an issue, this article presents a cost-effective IoT radon gas remote monitoring system able to obtain accurate concentration measurements. It can also trigger events to prevent dangerous situations and to warn users about them. Moreover, the proposed solution can activate mitigation devices (e.g., forced ventilation) to decrease radon gas concentration. In order to show its performance, the system was evaluated in three different scenarios corresponding to representative buildings in Galicia (Spain), a region where high radon gas concentrations are common due to the composition of the soil. In addition, the influence of using external hardware (i.e., WiFi transceivers and an embedded System-on-Chip (SoC)) next to the radon gas sensor is studied, concluding that, in the tested scenarios, they do not interfere with the measurements.

## 1. Introduction

Radon is a radioactive noble gas that is undetectable by the human senses, since it is odorless, tasteless and invisible. Moreover, under normal conditions, radon is also colorless, except when it cools down below its freezing point (that occurs at −71 ${}^{\circ }$C). Radon has its origin in nature, in the radioactive decay chain of uranium, which decomposes into lead. In such a decay chain, radium decomposes into radon, the most stable isotope of which has a half-life of 3.823 days [1] (half-life is the average time that a radioactive atom is expected to exist before it decays). Radon can also originate from the decomposition of thorium or actinium, but its half-life is only 55.6 s and 4.0 s, respectively [2]. In the aforementioned decay chains, under normal conditions, radon is gaseous, and therefore, it can be easily inhaled, being detrimental to human health. In fact, the effects of radon on health were first recorded in the 16th Century when observing the diseases suffered by miners in Eastern Europe, but it was not until the 1950s when it was determined that radon was the primary cause of cancer in miners [3]. Currently, the World Nuclear Association considers radon as the largest radiation contributor to humans [4], although its impact differs from one geological region to another.

The health effects derived from the presence of radon have a direct connection with the radioactive elements that arise from its disintegration, which are called the progeny of radon or radon daughters. Such elements, unlike gaseous radon, are solid and adhere to surfaces, just like the dust particles that travel by air. The main route of exposure to radon and its progeny is inhalation. Exposure to radon radiation is indirect. The danger to health does not come primarily from radon itself, but from radioactive products formed in the disintegration of radon. The general effects for the human body are caused by its radioactivity and the consequent risk of radiation-induced cancer.

Radon is a powerful pollutant that affects the quality of indoor air around the world. Epidemiological studies have shown a clear link between respiration of high concentrations of radon and the incidence of lung cancer [5]. Dust particles contaminated by radon daughters can also cause lung cancer. According to the World Health Organization, the exposure to radon is the second most common cause of lung cancer after smoking, and it is the primary cause of lung cancer among people who have never smoked [5]. Moreover, most of the radon-induced lung cancer cases occur among smokers due to a strong combined effect of smoking and radon.

The concentration of radon in the atmosphere is generally measured in Becquerel per cubic meter (Bq/m${}^{3}$) or in picocuries per liter (pCi/L). One pCi/L is equal to 37 Bq/m${}^{3}$. Radon gas concentration fluctuates between 5 and 15 Bq/m${}^{3}$ outdoors, while the values obtained indoors vary from 10–10,000 Bq/m${}^{3}$ [6], the soil and certain building materials usually considered as the dominating radon source [1]. In fact, radon is particularly common in regions with soils containing granite or slate, which have certain concentrations of uranium [7]. Despite its short half-life, radon gas can build up in buildings, especially in low-lying areas like basements because of its high density. It can also appear in groundwaters (i.e., in springs or hot springs), and its concentration may vary greatly depending on the season and the atmospheric conditions [1].

**Figure 1:**
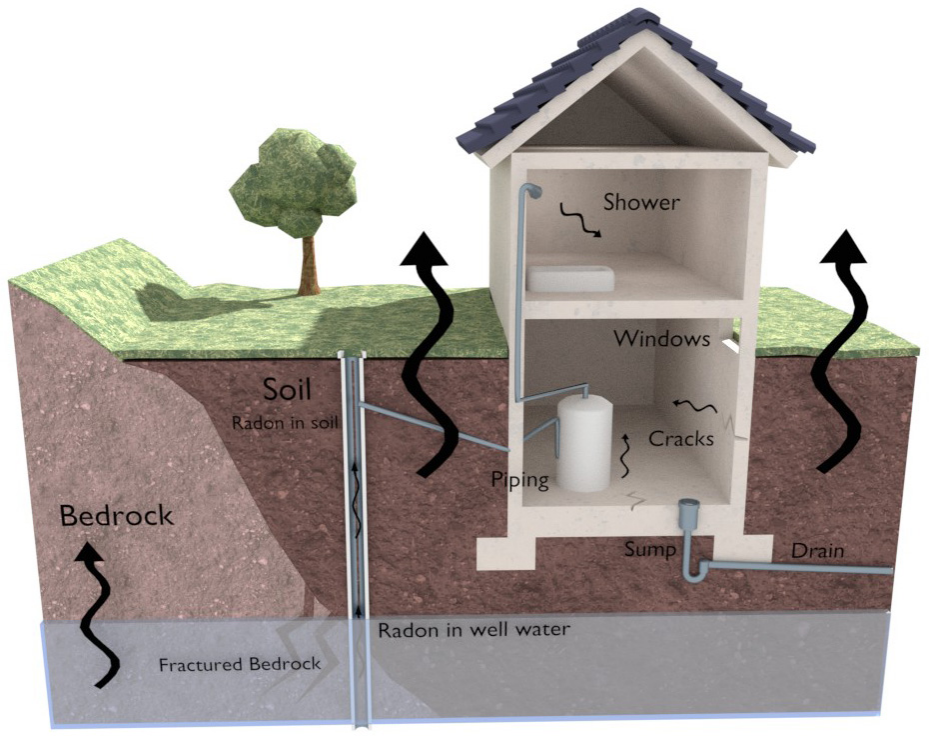
Possible radon entry points in a building.

Figure 1 shows the most relevant entry points of radon in a building. As can be observed, the black arrows illustrate how radon emanates naturally from the fractured bedrock either through the air or through underground waters (at the bottom of the figure). Thus, radon can access a building through piping, cracks in walls, holes in suspended floors, gaps around service pipelines, cavities within walls or water supplies. Since the most typical entry points in buildings are cracks, hermetically-sealed and poorly-ventilated buildings are the ones that suffer from the greatest risk of being exposed to radon.

The concentration of this gas in a building’s room can be significantly different from the concentration of a contiguous room. The average concentration ranges from less than 10 Bq/m${}^{3}$ to more than 100 Bq/m${}^{3}$ in some European countries. Note that the EPA (U.S. Environmental Protection Agency) recommends to take corrective measures for concentrations above 4 pCi/L (148 Bq/m${}^{3}$) [8]; in Canada the limit is 200 Bq/m${}^{3}$[9]; while the WHO sets such a level at 100 Bq/m${}^{3}$[5] (in countries where such a level cannot be reached due to their specific environmental conditions, the chosen reference level should not be over 300 Bq/m${}^{3}$). Since 2018, the EU members have established that the reference level should not exceed 300 Bq/m${}^{3}$ (according to EU Directive 2013/59/Euratom [10]).

Water from underground sources may contain significant amounts of radon depending on the conditions of the surrounding rock and soil, while surface sources generally do not [1]. Radon is also released from water as the temperature rises, the pressure decreases and when the water is aerated. In fact, the optimal conditions for the release of and exposure to radon occur when taking a shower. It is not known whether radon causes other cancers, but recent research suggests a positive association between such a gas and leukemia [11].

As a result of the combination of the latest advances in electronics, networking, computing and robotics, it is feasible today to develop low-cost advanced sensor systems. In the last few years, the application of the paradigm of the Internet of Things (IoT), together with other novel developments (i.e., industrial augmented reality [12,13,14], blockchain [15], smart devices [16]), has enabled performing and automating certain actions. IoT systems allow for carrying out enhanced decision-making in fields such as defense [17,18] and high-security applications [19], telemetry [20], transportation [21,22,23,24] or Industry 4.0 [25,26,27,28]. Among other applications, home automation or smart homes are rapidly gaining interest [29,30].

This paper presents a reliable, robust, cost-effective and scalable IoT system that is able to monitor the accumulation of radon gas remotely indoors remotely and, thus, prevents the above-mentioned health issues. The system has the following features:

- It is able to collect data from Commercial Off-The-Shelf (COTS) radon sensors and transmit them to a central server where they can be stored and processed.
- It offers a remote user-friendly web interface that shows the current and historic series of the radon concentration values.
- The system can be configured to set alert thresholds and sampling frequencies to react to abnormal radon gas concentrations.
- It is able to send remote alerts to the subscribed users to warn them about exceeding radon concentration levels.
- It can activate mitigation devices (e.g., forced ventilation) to decrease radon gas concentration.

The rest of this paper is structured as follows. Section 2 reviews the basics on radon gas detection and mitigation devices, as well as the most relevant academic and commercial systems. Section 3 details the architecture of the proposed system. Section 4 outlines the characteristics of the presented implementation. Section 5 describes the experimental setup and the results of the different tests performed. Finally, Section 6 is devoted to the conclusions.

## 2. Related Work

### 2.1. Types of Radon Detection Devices

There are different commercially available devices for the detection of radon [5]. The least expensive systems consist of short-term radon test kits (activated charcoal adsorption detectors), the collector of which has to be placed at the lowest floor of the house to be evaluated for a minimum of one day to up to a week. Then, the collector is sent by mail to a laboratory, where it is analyzed. Such activated charcoal adsorption kits, although affordable, have certain limitations. For instance, the obtained results are affected by humidity, so they have to be calibrated previously depending on the humidity range expected in the field. Moreover, since such a kind of detector enables continuous adsorption and desorption of radon, they only provide a good estimate of the average radon concentration if changes in radon concentration are small. Furthermore, the collector of the kit has to be returned as soon as possible, because the half-life of radon is only 3.8 days [31].

There are also long-term kits, which collect samples from one month to up to a year. These kits are called alpha-track detectors or solid state nuclear track detectors [32] and are based on a detecting material that is impacted by alpha particles, which produce microscopic areas of damage called latent alpha tracks. After the exposure period, such areas are counted either manually or automatically in a laboratory. The total number of tracks is proportional to the integrated radon concentration. Unlike activated charcoal adsorption devices, alpha-track detectors are not affected by humidity, but their results depend on altitude (especially above 2000 m), since air density can affect the distance that alpha particles can travel [33].

Digital detectors improve short- and long-term kits by providing average or continuous measurements that show daily, weekly, short-term and long-term average readings through a display. Such a kind of detector can be based on different detection principles, existing sensors that use electret ion chambers, scintillation cells, current or pulse ionization chambers or solid-state silicon detectors [5]:

- Sensors based on electret ion chambers: These are passive devices that act as integrating detectors that estimate the average radon gas concentration [34]. Such devices have shown excellent accuracy, but standard operation procedures must be followed to avoid interference from background gamma radiation [35] or the presence of dust in the electrets [36].
- Solid-state silicon detectors: Most electronic integrating devices are able to count the alpha particles emitted by radon and its decay products by making use of a solid-state silicon detector and a diffusion chamber. These devices have two main restrictions. First, they usually require long integration periods (more than two days), because of the small dimensions of their diffusion chambers. Second, their measurements can be altered in high humidity environments [37].
- Scintillation cells and current or pulse ionization chambers: These are mainly used for building continuous radon monitoring systems. Such systems collect air through a small pump and diffuse it into the sensing chamber, where measurements are performed during certain time intervals. Their features change depending on the sensing technique, but all of them usually share a common drawback: they require being calibrated periodically to guarantee accuracy and reliability.

Besides the detector type, several parameters should be taken into account when selecting a radon sensor. Accuracy is probably the most critical factor and the one that has a more relevant impact on price. Manufacturers usually indicate a number of Bq/m${}^{3}$ or a percentage of accuracy. Note that different parameters influence accuracy, both internal (e.g., related to electronics or internal technology) and external (i.e., environmental factors like relative humidity, air flow or temperature), so the specifications given by a manufacturer should be taken with caution.

Related to accuracy is the concept of the Lower Limit of Detection (LLD), which is the minimal amount of activity that can be detected by a radon sensor. LLD is essential when measuring low radon concentration levels and when establishing maximum thresholds, since a slight change might mean being over a reference level that can trigger certain events (e.g., the activation of an alarm).

Other relevant parameters are the sampling interval and the time required to show the initial measurements. Some detectors obtain a sample once a specific amount of time (e.g., once an hour), but others measure radon activity continuously. Moreover, some detectors are able to display initial concentration levels after just 48 h, but other sensor measurements can take up to several months.

Besides pricey industrial high-sensitivity radon monitors like [38], it is worth mentioning some of the few commercial radon home monitoring devices available, which differ in the previously-mentioned parameters. For instance, Coretium Home from Airthings [39] is probably the most advanced home radon monitoring system in terms of features. It is based on a low-cost (around €205 as of writing), low-power and easy-to-use sensing device that allows obtaining average short-term (last 24 h or last week) and long-term (since the last reset of the device) measurements.

Another home radon monitor is Rstone from Radiansa [40]. It is a compact reader with two buttons and a display, which also obtains information on other environmental factors like temperature, pressure and relative humidity. It can export the collected data to a PC and generate reports, as well as monitor and control the sensors remotely. It currently costs €1755.

The commercial radon monitor that has been modified to build the proposed IoT system is the Safety Siren Pro Series 3 [41]. It provides a digital display and is able to obtain short-term and long-term measurements. If the detected radon concentration level exceeds a threshold, the device can activate a sound alarm to warn the user. Its cost is roughly €120, and its main features are:

- It updates air samples and readings every hour.
- It provides short-term readings performed on the average of the last seven days.
- It collects long-term readings that are calculated as the average since the last reset of the device.
- It contains a menu button that toggles between short and long term. It also switches off the sound alarm and, when pressed during a certain amount of time, it restarts the sensor memory.
- To verify accuracy and reliability, the sensor auto-checks its internal state every 24 h.
- Guaranteed accuracy: ± 20% or ± 37 Bq/m${}^{3}$ (the highest of both).
- Typical accuracy: ± 10%.
- Sensor type: diffuse junction photodiode.
- It is actually small in comparison to other radon monitoring tools (its dimensions are 12.0 cm × 7.9 cm × 5.3 cm).

### 2.2. Academic Monitoring Systems

There are not many systems described in the literature explicitly aimed at monitoring radon gas concentration. For instance, a low-cost radon sensor is presented in [42]. In such a paper, the authors describe how they built and tested the monitoring device, which is based on a silicon chip that detects alpha particles produced during the decay of radon and its progeny. To be able to read the measurements remotely, the researchers embedded a wireless interface into the sensor.

Wireless communications can be also carried out by the monitoring system proposed in [43]. In such a case, a GPRS modem is used to transmit both radon concentration levels and the geographic position of the detector. The system was specifically devised for detecting very low radon gas concentrations with the help of a special ionic chamber and processing algorithms developed on a microcontroller (an ATmega16).

A different approach was performed by Shitashima et al. [44]: they focused on the detection of underwater radon for submarine applications. The authors proposed embedding a radon sensor into an Autonomous Underwater Vehicle (AUV) in order to collect measurements automatically for predicting earthquakes, volcanic activity monitoring, oceanography or hydrology. A similar development was presented in [45], but the monitoring system was aimed at geological prospections and environmental monitoring.

Other authors have focused on improving certain characteristics of the radon sensors. For example, Griffin et al. [46] built and tested a fast-responding sensor, which, according to their simulations, was able to reach 90% of its maximum count rate in 1.44 h. Another typical limitation of radon sensors is their range, which has been increased with the use of Long-Range Alpha Detector (LRAD) technology. A good example of such a technology is presented in [47], describing a simple, rugged and low-power detector that can be employed for continuous home radon monitoring.

Some researchers have also studied the performance of radon monitoring systems in different scenarios and environments. For instance, an interesting analysis was presented in [48], where the influence of electromagnetic interferences on a commercial radon sensor was analyzed. The paper relied on the fact that radon concentration is based on measuring very small currents (in the order of nano-amperes), which are very susceptible to electromagnetic perturbations, which may result in erroneous measurements. The researchers found relevant variations of the obtained measurements (with a difference of up to 375%) and thus demonstrated the influence of the electromagnetic interference from Ultra-High Frequency (UHF) sources in the 850–950-MHz band.

Other authors carried out radon measurements in different physical locations. One of them was performed in Transylvania (Romania) and analyzed radon concentration in surface waters, wells and springs with a commercial radon gas measuring kit [49]. The presented results showed relatively low levels of radon concentration (in surface waters) to medium (in spring waters), almost all samples collected in the study being safe to drink. The obtained results also showed a correlation between the concentration of radon gas and the geological structure of the terrain, finding the highest concentration values in regions where the soil was generally composed of granite formations.

A radioactive laboratory was the place chosen by Blanco et al. [50] to measure radon concentration levels. Specifically, the work described in the paper was aimed at reducing the interference of radon with different tasks performed in a measurement room of the lab. Such a measuring room was chosen because of its characteristics (small, closed and poorly ventilated), which made it ideal for finding high concentrations of radon gas. The researchers made use of an AlphaGUARD PQ 2000 PRO sensor [51], which is a portable device used for instantaneous or continuous measurements of radon gas concentration, both for short- and long-term tests in buildings and outdoors. The researchers found that the initial measurements gave a positive result in radon gas concentration, which led them to take mitigation measures. After discarding methods such as under-floor ventilation, sealing and drainage, the researchers decided to address the radon mitigation solution through positive ventilation, which consists of using fans that blow fresh and filtered air into the room, pressurizing to prevent radon gas from entering.

Finally, it is worth noting that other authors [52] opted for modeling radon gas concentration according to the values obtained previously during a measurement campaign. Based on their previous experience of collecting indoor radon concentrations in residential houses, the researchers proposed a prediction model to determine the potential indoor radon levels by taking into account parameters like the average indoor radon level in the area, the aerial radioactivity, the geology, the soil permeability or its structural type.

### 2.3. Mitigation Systems

There are different mitigation techniques that can be applied to reduce radon concentration. Some of them prevent radon from entering a building, while others decrease radon concentration after it has entered. The former ones are the ones recommended by the United States Environmental Protection Agency (U.S. EPA) [8]. The main ways for reducing home radon concentration are the following:

- Active soil suction (active sub-slab suction or sub-slab depressurization): This is considered the most reliable and common radon reduction method [8]. It consists of drawing the radon below a building through one or more pipes. Such pipes are inserted through the floor slab into the soil or rock underneath and allow for venting the radon to the air above the home. In homes where the foundations are built with hollow block walls, sub-slab suction can be used in conjunction with block-wall suction to depressurize the block wall. There is also a passive sub-slab suction variety that depends on natural air flows, but it is usually not as effective as active sub-slab suction when there is a high radon gas concentration.
- Perforated pipe, drain tile or sump-hole suction: These systems direct water away from the home foundations by suctioning it through pipes, tiles or by using sump pumps [53].
- Submembrane suction: This is a technique usually applied to crawlspace homes that consists of covering the earth floor with a high-density plastic sheet and then installing a pipe and a fan to draw the radon from under the sheet in order to vent it to the outdoors [54].
- Sealing cracks and openings in the foundations: Theoretically, this method prevents the radon from flowing into the building, but it has to be used in conjunction with another technique, since its use alone has not been shown to lower radon levels significantly.
- Home pressurization or use of air-to-air heat exchangers: These techniques consist of installing devices (a fan or heat exchangers) that blow outdoor air into the house. In the case of home pressurization, the incoming air creates pressure that prevents radon from entering the house, while air exchangers generate a heating or cooling air flow that helps to vent the house.
- Use of natural ventilation: Opening windows, doors and vents creates air flow that mixes outdoor and indoor air, which decreases radon concentration levels. However, note that natural ventilation is only a temporary solution, since it influences air conditioning and home security.

### 2.4. Analysis of the Related Work

After reviewing the state of the art on radon gas monitoring devices and the related work, the following conclusions were drawn:

- There are different commercial devices able to detect radon gas concentration, but they are either too expensive for the average householder (accurate readers are available from €1700), slow (e.g., long-term kits) or they do not provide Internet connectivity to access the collected values remotely (e.g., Rstone or Safety Siren Pro Series 3).
- Academic systems are a step ahead of commercial devices in terms of features, but it was found that none were explicitly designed as IoT devices.
- There is a wide range of mitigation systems to reduce radon gas concentration, but such systems are actually either passive approaches (e.g., sealing) or they are not usually connected to radon monitoring devices to act in a smart way.

As a result, in this paper, a system to tackle the three previous issues is presented: a low-cost IoT radon monitoring system is described, which is able to connect to mitigation systems in order to decrease radon gas concentration.

## 3. Design of the System

### 3.1. Architecture

**Figure 2:**
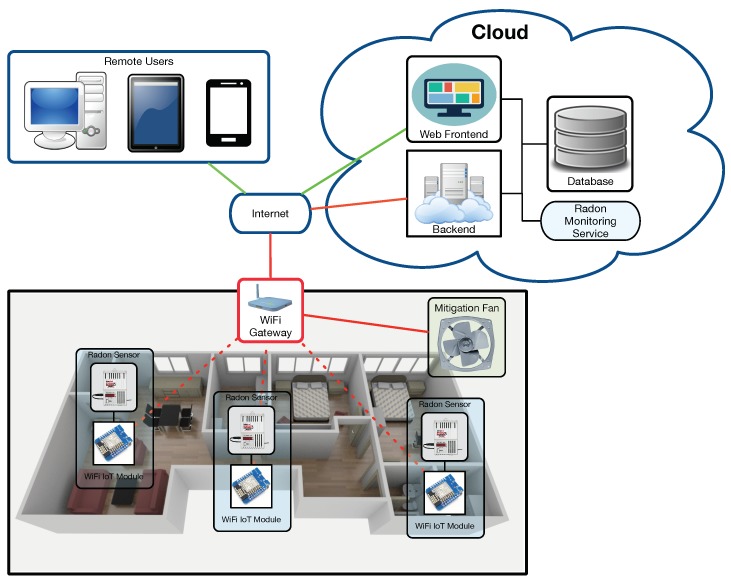
Communications architecture.

Figure 2 illustrates the communications architecture of the proposed system when deployed in a home. It can be observed that there is an internal home network where radon sensors collect measurements and send them wirelessly to a WiFi gateway. Then, the gateway sends such data to a cloud, where they are stored in a database (via a backend) and are made available to remote users that can access them through a web browser.

**Figure 3:**
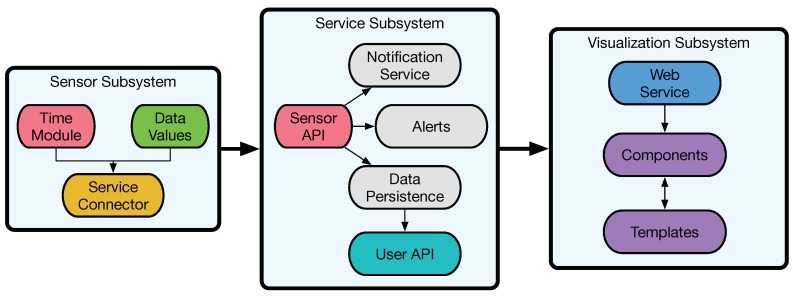
Subsystems that form the radon monitoring system.

Internally, the system is composed of the three subsystems depicted in Figure 3, which are described in the next subsections.

### 3.2. Sensor Subsystem

Each sensor node of the system consists basically of the previously-mentioned commercial sensing device (Safety Siren Pro Series 3) and a low-cost System-on-Chip (SoC). Specifically, the latter is an ESP8266 from Espressif Systems [55], which is 32-bit 80-MHz Tensilica Extensa MCU that provides WiFi connectivity through the embedded IEEE 802.11 b/g/n transceiver. The ESP8266 collects the data from the commercial radon sensor, assigns a timestamp to such data and sends them to the cloud.

The main challenge for this subsystem is that the commercial radon sensor does not include any communication interface, so it is necessary to modify it to capture the collected data. Such a modification enables intercepting the data sent to its display and requires emulating the actuation on a physical button.

**Figure 4:**
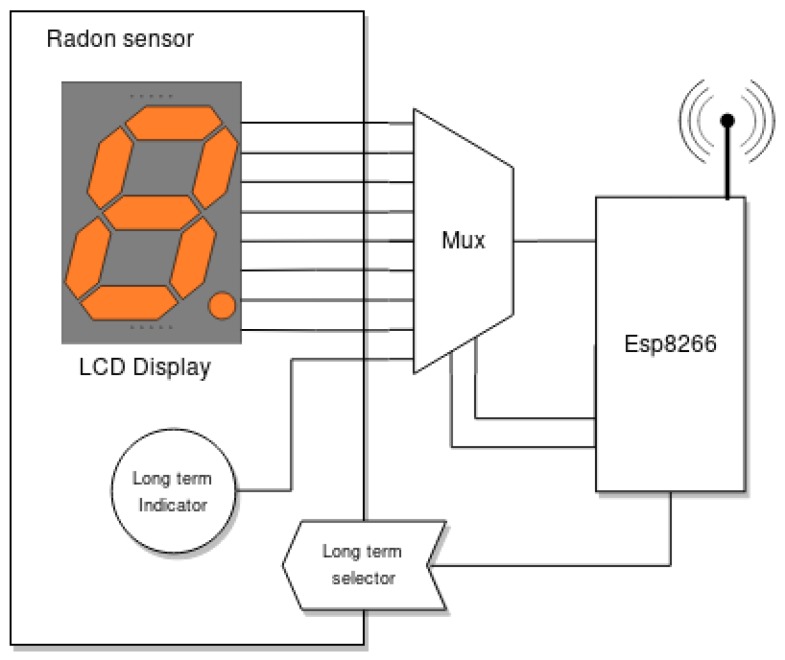
Interface with the sensor used to obtain the data.

**Figure 5:**
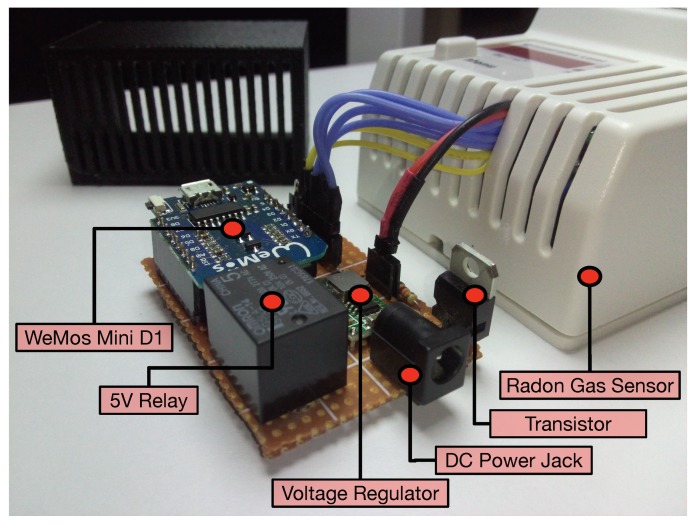
Components of the radon gas sensor.

Figure 4 illustrates how the ESP8266 and the radon sensor are connected, while Figure 5 shows part of such connections on the real hardware. Specifically, the following components can be distinguished in Figure 5 besides the commercial radon gas sensor:

- A WeMos Mini D1: This is a ESP8266-based board that provides USB-to-serial connectivity and multiple I/O pins to control other devices.
- A 5-V relay and a transistor: These are able to switch the radon sensor on and off remotely in case any problem arises.
- A voltage regulator and a DC power jack: These enable plugging in the official power adapter of the radon sensor (which works at 18 VDC) and stepping down the voltage to 5 V.
- A 16-to-one mux (CD74HC4067): This is actually inside the radon gas sensor. It is used because the display makes use of twelve parallel signals to communicate through a strobe encoding: seven of them are used for controlling the number shown in seven segments, and five strobe signals select the four digits. Since the number of input pins of the ESP8266 is reduced, the mux needs to be used as shown in Figure 4.

**Figure 6:**
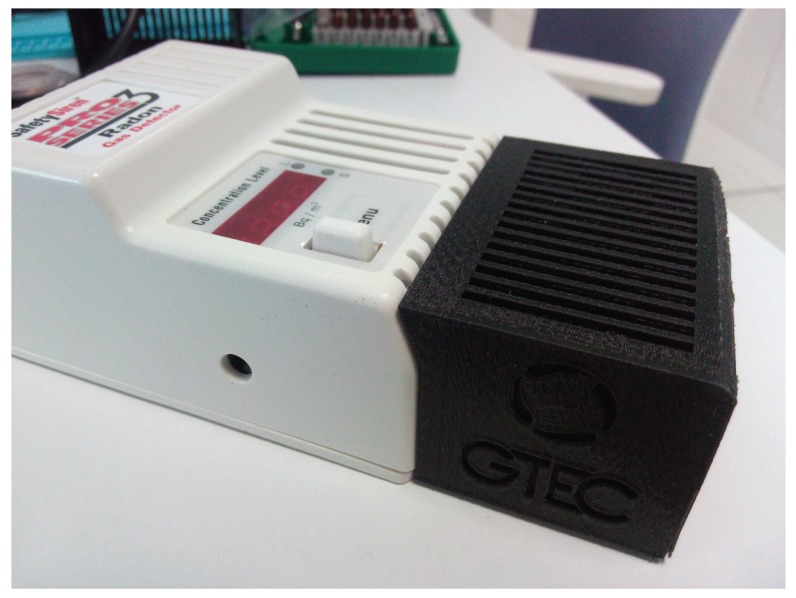
Radon sensor with the 3D-printed black box that contains the added electronics.

As can be observed in Figure 6, the radon sensor embeds a four-digit seven-segment-based display and a status LED that indicates if the values shown are related to short- or long-term measurements (a button to change between both measurement modes can also be observed in Figure 5 and Figure 6). All the additional hardware, except for the mux, is embedded into a 3D-printed black enclosure. Note that, as of writing, the cost of all the additional components is roughly €40.

Since the radon sensor updates its display once an hour, the ESP8266 can be programmed to enter into deep-sleep mode to reduce power consumption. In deep-sleep mode, the wireless transceiver and most hardware are turned off except for the Real-Time Clock (RTC), which is the one responsible for waking the SoC up every hour. Note that the ESP8266 can consume up to 240 mA, but in deep-sleep mode, it only draws 20 μA.

Regarding the timestamping of the data, it is worth mentioning that the system is updated via the Network Time Protocol (NTP) everyday or when it boots up, in order to maintain time synchronization. **This timestamping is essential for keeping the traceability of the measurements since it allows the** system to preserve timing if there are connectivity problems or network restrictions when transmitting the collected data.

### 3.3. Service Subsystem

This subsystem is responsible for providing two APIs related to the communications with the sensors (SensorAPI) and with the users (UserAPI). The Sensor API enables setting up the different sensor node parameters, while the User API allows for easing the interaction with the user, including how the information is displayed or the configuration of alarms and thresholds.

The service system can be accessed by different users simultaneously, so every user is able to add new sensors and operate them independently. In the same way, many sensors can report their data to the service subsystem, so they are identified with unique tokens.

Internally, this subsystem makes use of MongoDB [56], which is a non-relational document-oriented database. In the data model, the data collected by a specific sensor during a day are embedded into a document, so 24 values are provided. In addition, the mean, maximum and minimum values of the day are added.

Inside the service subsystem, there is a specific sub-service (the notification service) that is aimed at detecting when the radon concentration levels exceed the prefixed threshold. In such a case, the subsystem is able to trigger certain actions, which usually consist of warning the user or other systems. Currently, the system sends Telegrammessages to a mobile phone number indicated by the user in the configuration settings.

### 3.4. Visualization Subsystem

The visualization subsystem provides a user-friendly interface that plots the current and past radon concentration values. It also eases user interaction with the configuration and events detected by the system.

**Figure 7:**
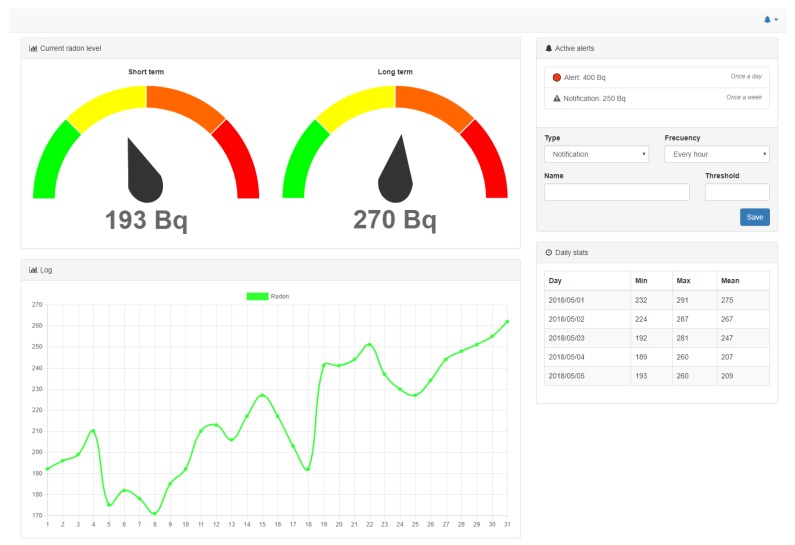
Dashboard of the radon gas monitoring system.

Specifically, the user interface of the visualization subsystem (in Figure 7) was designed as a web dashboard. The interface collects the data to be shown in an asynchronous way from the service subsystem and plots in real time the short- and long-term radon concentration values. In addition, the interface enables configuring the prefixed thresholds that are monitored by the notification service.

### 3.5. Communication Protocols

**Figure 8:**
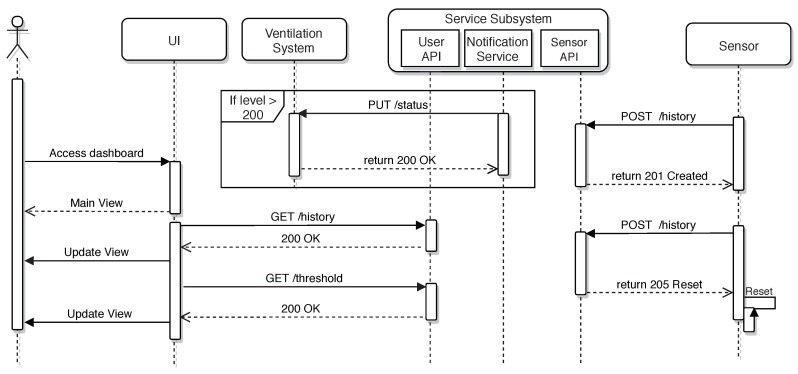
Sequence diagram about the sensor value collection and user interaction.

The sequence diagram depicted in Figure 8 summarizes the main interactions that can be performed in the system. On the right of Figure 8, it can be observed how the radon gas sensor interacts with the Sensor API. The sensor posts periodically (by default, once an hour) the collected values, and the API acknowledges the reception with a 201 code. If the sensor needs to be reset by the user, the Sensor API returns 205. Note that this way of indicating the reset is required because the sensor remains asleep most of the time, and it only wakes up to collect and send the measured values.

Figure 8 also shows how the mitigation measures are taken. The notification service monitors the collected values, and when they exceed a prefixed threshold (in the Figure 8, such a threshold is 200 Bq/m${}^{3}$), this is communicated to the ventilation system, which actuates the mitigation fans.

The third main interaction illustrated in Figure 8 is related to how the user interacts with the web dashboards. As can be observed, the user first accesses the main view of the dashboard, but periodically, such a view is updated as data are collected from the sensors and as different events are notified.

## 4. Implementation

### 4.1. Implemented Features

Although the main objective of the proposed system is to provide the collected radon sensor values to remote users, additional features have been added to extend its functionality and flexibility. Specifically, the following features are included:

- It shows the current short- and long-term radon gas concentration values.
- It plots the historical evolution of the concentration of radon in order to detect trends.
- It is possible to add alerts to the system depending on a threshold that indicates the minimum radon gas concentration. Once the threshold is exceeded, an alert is notified to the user. The configured alerts can be checked with a certain frequency, allowing for sending notifications once an hour, once a day, once a week or once a month.
- Alerts are shown in a specific section of the web dashboards, but they can also be notified to the user through different communication systems (e.g., email, Telegram, SMS).
- The dashboard also shows relevant daily statistics like the mean, minimum and maximum radon gas concentration values.
- It allows for adding multiple radon sensors to the system so that different users can access them in parallel.
- Sensor nodes can be configured remotely, so a technician does not have to go to the place where the sensor is installed to reprogram the firmware.
- Two APIs are provided (Sensor and User API) so that third-parties can interact with the system and develop their own software and add new features to the system.

### 4.2. Implemented APIs

#### 4.2.1. Sensor API

The SensorAPI is responsible for the communications between the radon sensor and the service subsystem. Such an interface has write permissions in only one direction because the responsibility of the sensors is to store radon concentration data. This task is performed through a POST request to the endpoint*/history*, which is responsible for storing the measurement history.

After saving the new measurement, the response usually issued by the service is *201 Created*, but depending on the sequence of values previously sent, anomalies in the sensor can be detected. In such a case, after saving the value, the service would return *205 Reset content*, to request a reset of the sensor to the microcontroller (the reset consists of switching the power supply of the sensor on and off by using the embedded relay).

#### 4.2.2. User API

The UserAPI is responsible for the communications between the user interface and the service subsystem. All tasks related to visualization and configuration are carried out through this API, whose main functions are:

- GET*/history* allows obtaining the stored values in a given time range. Three parameters are required: the sensor identifier (SensorID) and the start and end dates.
- GET*/threshold* allows obtaining the configured alert levels that trigger the different mitigation actions. There are also DELETE and POST methods on this endpoint, which make it possible to eliminate and create new alerts, respectively.

### 4.3. Visualization and Control Software

In order to implement the remote visualization system, a web dashboard has been developed, in which the history of data obtained by the sensor can be seen, as well as the configuration of alerts and actions associated with them.

This application has been developed in a modular way so that each of the modules have the responsibility of obtaining the necessary information and of processing it properly to show it to the user. Each module works asynchronously and is responsible from drawing its part of the visual interface. In Figure 8, some of the asynchronous requests issued from each of the modules, as well as how the interface is updated accordingly can be seen.

### 4.4. Notification Service

This service is responsible for carrying out actions based on the configured alerts. The communication interface changes depending on the type of action configured for each one of the alerts. For instance, the proposed system is able to send a notification via a Telegram bot. Such a notification triggers a POST request to the Telegram API with the description of the alert. Another action could consist of activating the ventilation system by means of an HTTP request to the system controller.

**Figure 9:**
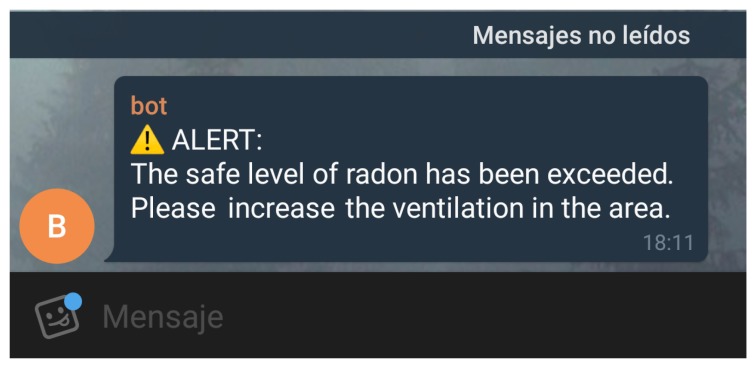
Telegram notification about radon gas concentration.

As an example, Figure 9 shows a Telegram notification that warns about an exceeded radon level in one of the test locations evaluated in Section 5.

## 5. Experiments

### 5.1. Radon Gas Concentration

**Figure 10:**
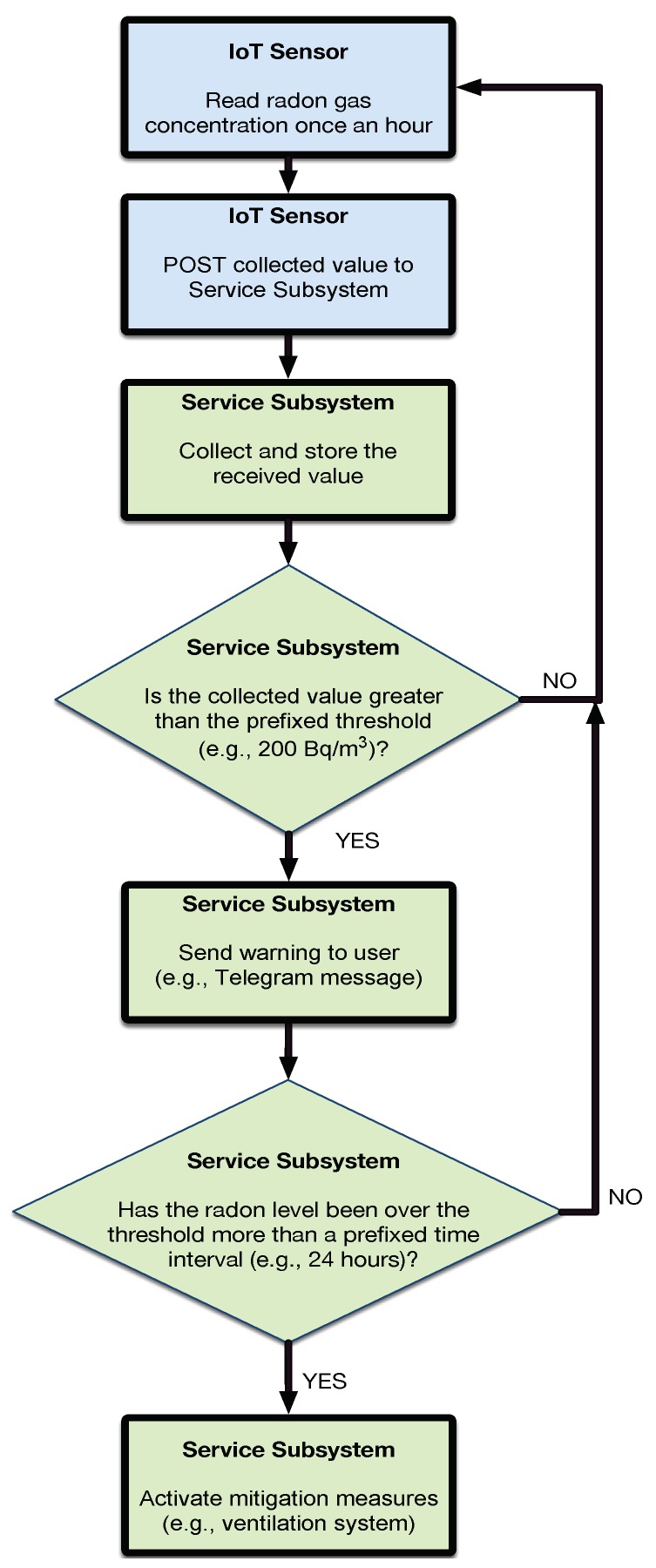
Control flow of the tested system.

The proposed radon gas monitoring system was evaluated in three different scenarios in Galicia (Spain): an urban home, a traditional Galician country house and a research lab. These three scenarios are considered as representative of the types of households and working buildings, but it is important to emphasize that radon gas concentration varies dramatically from one area to another, and even in the same area due to the different factors mentioned previously in Section 1. In the three selected scenarios, the control flow was modeled as illustrated in Figure 10. Sensor data were collected and stored once an hour. If a received value exceeded a prefixed threshold (e.g., 200 Bq/m${}^{3}$), the system warned a predetermined user (for instance, by sending a Telegram message). If the collected values exceeded the prefixed threshold during a certain amount of time (which can be configured), mitigation measures were taken (e.g., activating the forced ventilation). However, note that, in order to avoid interfering with the experiments described next, which analyzed how radon gas fluctuated naturally, the mitigation measures were actually not enabled.

Note also that, for the sake of brevity, only short-term values were plotted since they measure the average of the past seven days, allowing the user to monitor short-term fluctuations and detect seasonal and weather-related variations in radon levels.

Finally, it is worth pointing out that, despite the sensor accuracy claimed by the manufacturer, in some scenarios, it may not be enough. Note also that there are standard recommendations for performing standard radon concentration measurements [57,58], and although the selected sensor was approved by the U.S. Environmental Protection Agency, Radiation and Indoor Environments National Laboratory, the accuracy requirements may change through time and from one country to another. For such cases, it is possible to calibrate the sensor with high-sensitivity radon detectors and then remarkably improve the measurements obtained by the Safety Siren Pro Series 3 [59]. In the case of having doubts when evaluating an environment with the Safety Siren Pro Series 3, it would be recommended to perform measurements for at least a week (it seems that this is enough time to obtain stabilized measurements from the Safety Siren Pro Series 3 in a static environment), and then, if radon gas concentrations near the maximum threshold are detected, a more detailed analysis with accurate devices would have to be performed before taking mitigation measures. Therefore, the proposed system would be used more like a screening system to get an initial understanding of the environment before follow-up tests or mitigation measures.

#### 5.1.1. Urban Home

The first environment to be analyzed consisted of a flat on the fourth floor of an urban building in the city of A Coruña. The front of the building was not made out of granite or slate, although they are present in many buildings of the area. Inside the flat to be monitored, there was only a potential source of radon: the kitchen counter, which was made out of granite.

**Figure 11:**
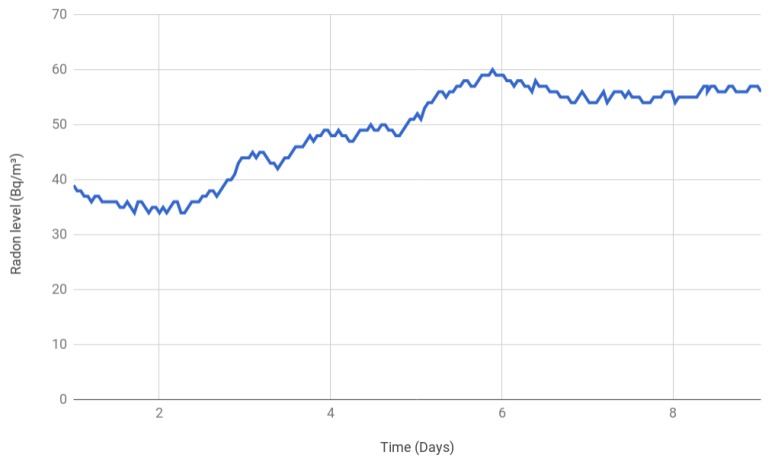
Radon gas concentration in an urban home.

To perform the tests, a room with a low-medium ventilation frequency was selected. The ventilation consisted basically of opening the windows in different rooms to create a natural air flow. In such a scenario, the values shown in Figure 11 were obtained during a nine-day period.

It can be observed that, during the first three days, the values were under 40 Bq/m${}^{3}$, but then radon gas started to accumulate until it reached 60 Bq/m${}^{3}$, where it remained stable. Despite the observed accumulation, the radon gas concentrations were below the thresholds indicated by local and international authorities, so it can be concluded that, while the scenario was monitored, no concentration issues were detected.

#### 5.1.2. Galician Rural Home

For the second scenario, a traditional Galician rural home was selected. It is a three-story home whose basement and first floor walls are made out of granitic stones. The facing of the house walls is also made out of granite. Inside the house, the kitchen counter and the stairs flooring are made out of granite.

**Figure 12:**
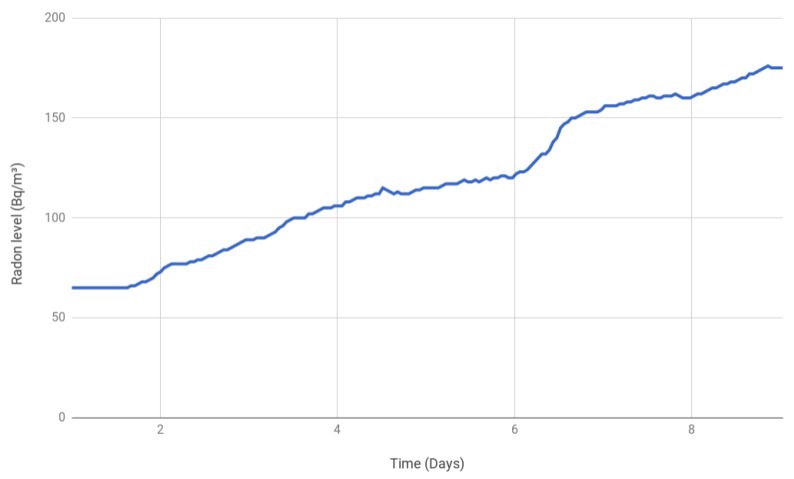
Radon gas concentration in a rural home.

The radon gas concentration was measured in a room of the basement, which only had a small window. Figure 12 shows that the measurements started around 60 Bq/m${}^{3}$, but then, as the days went by, radon accumulated until it reached around 175 Bq/m${}^{3}$. Such a maximum value is below the EU’s and Canada’s maximum thresholds that require taking preventive measures (300 and 200 Bq/m${}^{3}$, respectively), but it is over the EPA’s and WHO limits (148 and 100 Bq/m${}^{3}$). Nonetheless, please note that the Safety Siren Pro Series 3 accuracy is guaranteed to be ±20% or 37 Bq/m${}^{3}$ (the larger of both), so for a reference level of 300 Bq/m${}^{3}$, in the most conservative case, the threshold should actually be set at 240 Bq/m${}^{3}$. In any case, since the trend towards accumulation was clear, it was recommended to ventilate frequently through natural or artificial air flows and to continue to monitor the house.

#### 5.1.3. Research Lab

The third scenario was set up in the Group of Electronic Technology and Communications (GTEC) laboratory of the University of A Coruña. The lab is on the second floor in a recently-constructed building that is essentially made out of reinforced concrete. Nonetheless, the building is on a campus where high radon gas concentrations have been detected in the last few years, and even some parts of the faculties have been closed due to the detection of high values of radon gas concentration.

**Figure 13:**
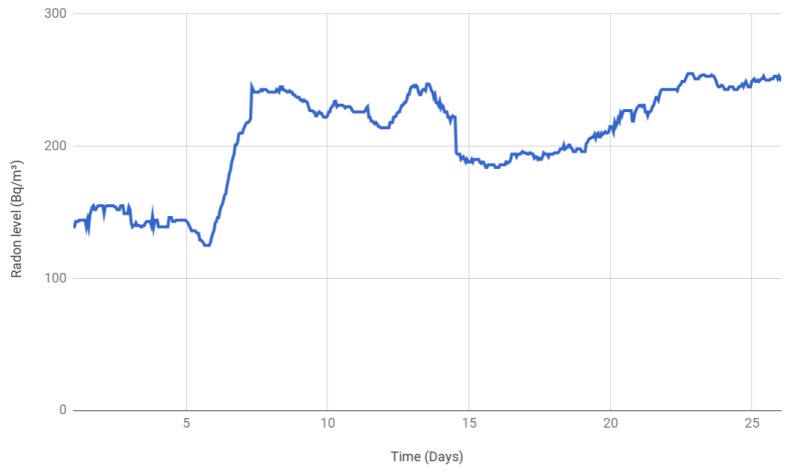
Radon gas concentration over 26 consecutive days.

In such a scenario, the sensor was first placed in a specific location for a month to stabilize its measurements. Then, data were collected during the next 26 consecutive days of the same month, performing 24 measurements per day. The results are shown in Figure 13. It can be observed that, at the beginning of the month, the values were around 150 Bq/m${}^{3}$, but from Day 7, they increased significantly and even exceeded 250 Bq/m${}^{3}$. In fact, the measured mean radon level for the 26 days was 201.8 Bq/m${}^{3}$.

The collected values remained most of the time over the limits established by the WHO (100 Bq/m${}^{3}$), the EPA (148 Bq/m${}^{3}$) and the Canadian authorities (200 Bq/m${}^{3}$). However, during the whole measurement period, it did not exceed the threshold indicated by the European Union (300 Bq/m${}^{3}$). In any case, due to the clear trend towards radon gas accumulation, it was recommended to ventilate the lab often.

### 5.2. Interference Analysis

Recently, it was detected that the measurements performed with the commercial radon gas sensor used in the proposed system were altered by UHF wireless transceivers [48]. Since in the present article, the use of a WiFi module to collect the sensor values was proposed, different experiments were carried out to determine whether such a module influenced the measurements.

#### 5.2.1. WiFi Interference under Regular Use

The first experiment consisted of comparing the measurements obtained by two sensors that were close to each other: one of them was not modified (i.e., it was used as sold), while the other one embedded in the SoC and with WiFi was enabled (i.e., it transmitted the collect measurements once per hour to the remote monitoring service). The values of the unaltered sensor were collected manually by the lab technicians (i.e., they wrote down the value shown on the sensor display).

**Figure 14:**
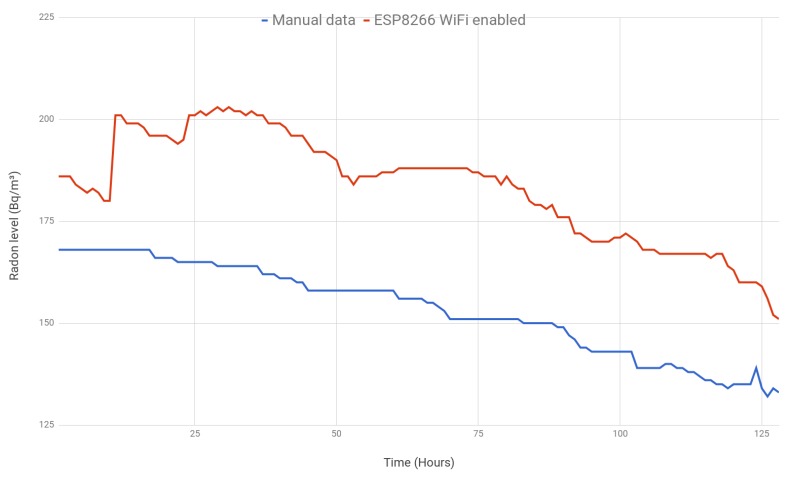
Manual data (without ESP) vs. ESP with WiFi enabled.

The obtained results are shown in Figure 14. The line in red represents the values collected by the WiFi-enabled sensor, while the line in blue is for the unmodified sensor. A high positive correlation (0.890) between both measurements is observed, especially after the first 48 h (correlation increases to 0.956). Note that, according to the manufacturer, the selected radon gas sensor, when placed in a new location, requires 48 h to stabilize its measurements since it needs a minimal amount of samples to average the results.

Although the range of the results varies (the WiFi-enabled sensor values ranged from 151–203 Bq/m${}^{3}$, while the unmodified sensor values ranged from 132–168), the average difference is 30.06 Bq/m${}^{3}$. Note that the manufacturer’s guaranteed accuracy is ± 20% or 37 Bq/m${}^{3}$ (the highest of both). After a month, the sensors converged to similar values, and the difference between both was less than 10 Bq/m${}^{3}$. Therefore, it can be concluded that, despite there being certain differences between both sensors, there were no clear oscillations after the first 48 h that would involve the existence of interference from the use of the embedded SoC when using WiFi as proposed in this article.

#### 5.2.2. SoC Interference

A second experiment was performed to determine whether the installed SoC might interfere with the collected values. For such an experiment, two radon gas sensors were modified to be embedded in ESP8266 SoCs, but one of them enabled the WiFi module (transmitting once per hour), while the other one disabled wireless communications.

**Figure 15:**
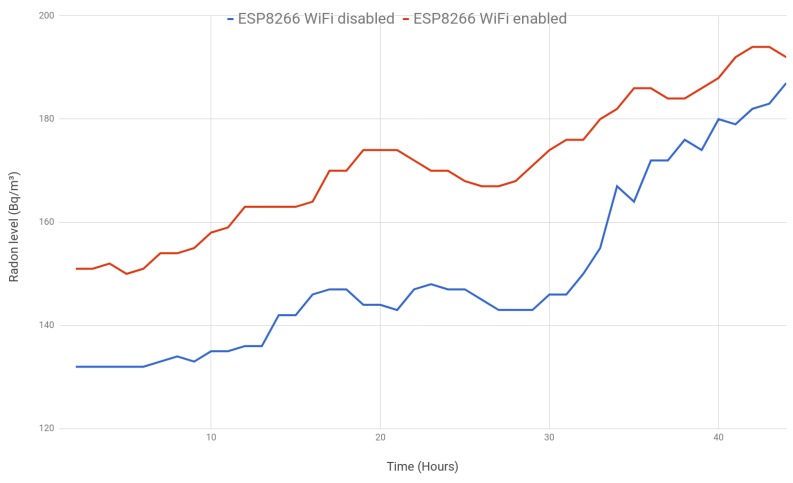
ESP8266 with WiFi enabled vs. ESP8266 with WiFi disabled.

Figure 15 shows the evolution of the two sensors. Like in the previous experiment, a high positive correlation (0.935) between both measurements is observed. In this case, the average absolute difference is 20.32 Bq/m${}^{3}$, but after the first 48 h it goes down to 11.27 Bq/m${}^{3}$.

In view of the results, no relevant differences were observed on the collected measurements as a consequence of the use of the WiFi module.

#### 5.2.3. High-Traffic WiFi

The last experiment was aimed at determining whether the continuous use of WiFi might alter the measured values. While in the previous experiments, the collected values were transmitted once per hour (recommended to save power), in this test, it was decided that the system would make continuous HTTP requests to a remote Internet web server in order to add more traffic (and, consequently, more WiFi signals) to the scenario. The requests consisted of requesting and downloading the content of two web pages each minute (about 3 MB per minute). In this case, while one of the radon sensors was generating additional WiFi traffic, the other sensor embedded the SoC, but disabled its WiFi module (the collected values were first stored in the Flash memory and then dumped to a PC).

**Figure 16:**
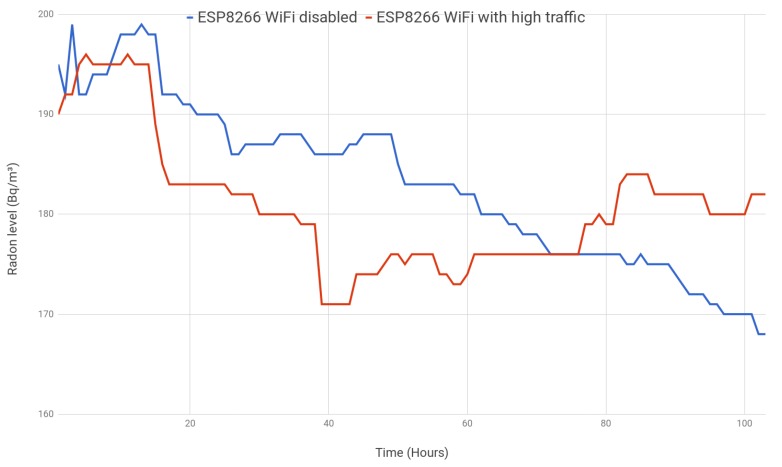
ESP8266 with WiFi disabled vs. ESP8266 with WiFi enabled and high traffic.

The obtained results are shown in Figure 16. It can be observed that they stay pretty close for the entire time that they were monitored. In fact, their mean value is almost the same (approximately 180 Bq/m${}^{3}$), and the variance of the absolute difference between both measurements is only 16.38 Bq/m${}^{3}$ (i.e., the standard deviation is roughly just 4 Bq/m${}^{3}$). As a consequence of the previous results, we conclude that there is not a clear influence of the high WiFi traffic on the sensed values.

## 6. Conclusions

This article presented a cost-effective IoT radon gas monitoring system. It is less expensive than solutions with similar accuracy, but no IoT features (e.g., Coretium Home, €205), and than more accurate, but less flexible devices with less features, like Rstone (€1755), AlphaGUARD PQ 2000 Pro (€9700) or RAD7 (€4260). The system is based on a commercial radon gas sensor modified to be embedded in an SoC able to provide additional intelligence and wireless communications. The system is able to monitor the sensed values continuously and show them to remote users through a web dashboard. In addition, the system is able to send alerts to such users, and it can activate mitigation devices (e.g., forced ventilation) to decrease radon gas concentration in a smart way in the case of detecting abnormal values. The system was evaluated in three different scenarios in Galicia (Spain), which showed different radon gas concentration values and trends. Finally, diverse tests were performed in order to determine if the embedded electronics and the wireless communication module might influence the collected values, but the results showed that there were no signs of interference.

To sum up, all the obtained results confirm that the system provides a good tool for informing the users about the radon levels at their home. The availability of real-time data enables the use of mitigation techniques to avoid the prolonged exposure to high concentrations of this radioactive gas.

## Abbreviations

API	Application Programming Interface
AUV	Autonomous Underwater Vehicle
COTS	Commercial Off-The-Shelf
LLD	Lower Limit of Detection
LRAD	Long-Range Alpha Detector
NTP	Network Time Protocol
REST	REpresentational State Transfer
RTC	Real-Time Clock
SoC	System-on-Chip
UHF	Ultra-High Frequency
WHO	World Health Organization
